# Development of a Rapid Cartilage Damage Quantification Method for the Lateral Tibiofemoral Compartment Using Magnetic Resonance Images: Data from the Osteoarthritis Initiative

**DOI:** 10.1155/2015/634275

**Published:** 2015-12-02

**Authors:** Ming Zhang, Jeffrey B. Driban, Lori Lyn Price, Grace H. Lo, Eric Miller, Timothy E. McAlindon

**Affiliations:** ^1^Division of Rheumatology, Tufts Medical Center, 800 Washington Street, P.O. Box 406, Boston, MA 02111, USA; ^2^The Institute for Clinical Research and Health Policy Studies, 800 Washington Street, P.O. Box 63, Boston, MA 02111, USA; ^3^Tufts Medical Center and Tufts Clinical and Translational Science Institute, Tufts University, 800 Washington Street, P.O. Box 63, Boston, MA 02111, USA; ^4^Medical Care Line and Research Care Line, Houston Health Services Research and Development (HSR&D) Center of Excellence Michael E. DeBakey VAMC, Houston, TX 77030, USA; ^5^Section of Immunology, Allergy, and Rheumatology, Baylor College of Medicine, 1 Baylor Plaza, BCM-285, Houston, TX 77030, USA; ^6^Department of Electrical and Computer Engineering, Tufts University, 216 Halligan Hall, Medford, MA 02155, USA

## Abstract

The purpose of this study was to expand and validate the cartilage damage index (CDI) to detect cartilage damage in the* lateral* tibiofemoral compartment. We used an iterative 3-step process to develop and validate the lateral CDI: development (100 knees), testing (80 knees), and validation (100 knees). The validation set included 100 knees from the Osteoarthritis Initiative that was enriched to include all grades of lateral joint space narrowing (JSN, 0–3). Measurement of the CDI was rapid at 7.4 (s.d. 0.73) minutes per knee pair (baseline and follow-up of one knee). The intratester reliability is good (intraclass correlation coefficient (3, 1 model) = 0.86 to 0.98). At baseline, knees with greater KL grade and lateral JSN had a lower mean CDI (i.e., greater cartilage damage). Baseline lateral CDI is associated with both lateral JSW (*r* = 0.81 to 0.85, *p* < 0.01) and HKA (*r* = −0.30 to −0.33, *p* < 0.05). The SRM is good (lateral femur SRM = −0.76; lateral tibia SRM = −0.73; lateral tibiofemoral total SRM = −0.87). The lateral tibiofemoral CDI quantification allows for rapid evaluation and is reliable and responsive, with good construct validity. It may be an efficient method to measure lateral tibiofemoral articular cartilage in large clinical and epidemiologic studies.

## 1. Introduction

Cartilage morphometry on magnetic resonance (MR) images is important for the assessment of structural progression of knee osteoarthritis (OA). However, manually obtaining accurate and reproducible cartilage data on one set of images can take many hours [1]. To reduce the time and cost of measuring cartilage on MR images, there remains a great need to design a rapid quantification method which has good reproducibility, validity, and sensitivity to change [2].

In our previous study, we developed the cartilage damage index (CDI) for the* medial* knee compartment and demonstrated it to be an efficient, reliable, valid, and sensitive method to measure changes of articular cartilage in the* medial* tibiofemoral compartment. This new study builds on our previously published paper by adapting and testing the CDI to the* lateral* tibiofemoral compartment. It is important to note that we needed to modify the CDI because the medial and lateral tibiofemoral compartments have different articular surface shape [3], loading [4], and distributions of full thickness cartilage defects [5]. Because of the differences between compartments, we needed to identify new informative locations that are specific to the lateral tibiofemoral compartment and test whether these new locations could offer an efficient, reliable, valid, and sensitive method to measure changes of articular cartilage in the lateral tibiofemoral compartment. Hence, the purpose of this study was to adapt and validate the CDI to detect cartilage damage in the lateral tibiofemoral compartment.

## 2. Methods

### 2.1. Study Design

We developed, validated, and assessed reliability of the CDI in the lateral tibiofemoral compartment. Sampling from the Osteoarthritis Initiative (OAI), we created 4 datasets: (1) a development dataset (*n* = 100 knees), (2) a test dataset (*n* = 80 knees), (3) a validation dataset (*n* = 100 knees), and (4) a reliability dataset (*n* = 20 knees).

### 2.2. MR Image Assessments

To deploy the CDI, we focused on the OAI 3D sagittal water-excitation dual-echo steady state (DESS) images, which were acquired using the OAI MR imaging protocol [6]. The OAI has institutional review board approval (IRB) from the coordinating centers and the four clinical centers (University of Maryland and Johns Hopkins comprise a single recruitment center, Brown University, Ohio State University, and University of Pittsburgh). All participants provided informed consent to participate in the OAI. The 3D DESS sequences were acquired using the following parameters: field of view = 140 mm, slice thickness = 0.7 mm, skip = 0 mm, flip angle = 25 degrees, echo time = 4.7 ms, recovery time = 16.3 ms, 307 × 384 matrix, *x* resolution = 0.365 mm, *y* resolution = 0.456 mm, and total slice number = 160. The acquisition time for 3D DESS sequence is 11 minutes.

### 2.3. Development Dataset

**Figure 1:**
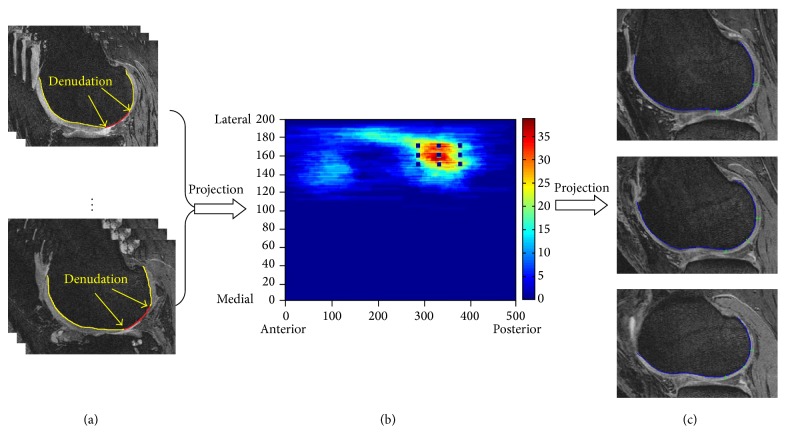
(a) Manual lateral femur cartilage mark. (b) Heat map of lateral femur denudation projection. (c) Lateral femur CDI measurement on 3 selected slices.

For the development dataset, we selected 100 knees from OAI baseline that included an equivalent number of knees with the different grades of lateral joint space narrowing (JSN, grades 0–3). We used three steps to develop the lateral tibiofemoral CDI based on areas commonly affected by denudation. (1) One reader manually marked the lateral cartilage denudation on each knee (Figure 1(a)). (2) We designed a pair of two-dimensional, rectangular, universal coordinate systems to represent the articular surface on the distal lateral femur and the proximal lateral tibia (Figure 1(b)). (3) We projected the regions of denudation onto a coordinate system and constructed a figure illustrating the frequency distribution of denudation in a three-dimensional representation of the lateral compartment. We used this to evenly select 9 informative locations on the tibia and femur (18 locations in total) in and around the regions that most frequently exhibited denudation (Figure 1(b)). We hypothesize that this region has more frequent cartilage damage.

### 2.4. Lateral Tibiofemoral CDI Measurement

There are three steps to measure the lateral CDI. (1) The reader determines the medial-lateral width of the femur by selecting the most medial and lateral MR image slices possessing bone. These images represent the *y*-axis (medial-to-lateral) of the coordinate system (Figure 1(b)). The software automatically indicates the slices that contain the informative locations based on the coordinate system. (2) The reader manually marks the bone-cartilage boundary on the selected slices (Figure 1(c)). The software then projects the bone-cartilage to *x*-axis (anterior-to-posterior) of coordinate system and indicates the predefined informative location on the MR slices. (3) The reader measures the cartilage thickness at those informative locations (Figure 1(c)). The software then computed the CDI by summing the products of cartilage thickness, cartilage length (anterior-posterior), and voxel size from each informative location. To normalize for body size, the CDI for the lateral tibia and femur was divided by the individual's height.

### 2.5. Test Dataset

We performed preliminary tests to explore face and construct validity by selecting 80 participants from the OAI. These 80 knees all had publicly available manual cartilage segmentation on baseline and 12-month follow-up MR images (Imorphics Ltd; the dataset originally included 88 knees but we excluded 8 knees with missing height or hip-knee-ankle (HKA) angle). These participants also had height data available at each visit. One reader used customized software to measure the CDI in the lateral femur and tibia cartilage in the testing dataset.

### 2.6. Validation Dataset

To test the validity of the lateral tibiofemoral CDI—the main purpose of this study—we selected 100 knees with baseline and 24-month MR images from the OAI. The validation samples were chosen to represent a wide range of disease severity. The dataset was selected to include all grades of lateral JSN (*n* = 25 knees per lateral JSN grade) and knees with and without lateral JSN progression (JSN grade change between baseline and follow-up visit). None of these knees was included in the development or test datasets. The first ten ids were used to record the measurement time.

### 2.7. Reliability Dataset

In addition to the final validation set, we identified 20 other knees to assess intratester reliability (two measurements separated by at least 72 hours). The reliability set was selected based on baseline lateral JSN grade (5 knees per lateral JSN grade).

### 2.8. Radiographic Assessments

Participants had bilateral weight-bearing, posterior-anterior, semiflexed knee radiographs at each annual OAI visit. Central readers provided Kellgren-Lawrence (KL) grade and the modified OARSI-atlas based assessment of lateral JSN score [7, 8]. The radiographs, central readings, and protocols are publicly available at the OAI website (kxr_sq_bu_00 (version 0.5) and kxr_sq_bu_03 (version 3.5); http://oai.epi-ucsf.org/; reliability for these readings was kappa = 0.70 to 0.88).

The same bilateral knee radiographs were also used to provide central measurements of lateral tibiofemoral joint space width (JSW). We selected lateral JSW at one fixed location (*x* = 0.725). JSW data and descriptions of the methods are publicly available on the OAI website (kxr_qjsw_duryea_00 (version 0.5) and kxr_qjsw_duryea_03 (version 3.4); http://oai.epi-ucsf.org/; reliability for these readings was ICC > 0.93).

Finally, we used publicly available measures of static alignment (HKA angle) that was measured by a third investigator. The HKA angles were measured on full limb films primarily at the 12-month or 24-month OAI visits. The HKA data and descriptions of the methods are publicly available on the OAI website (flXR_KneeAlign_Cooke01 (version 1.2) and flXR_KneeAlign_Cooke03 (version 3.1); http://oai.epi-ucsf.org/; reliability for these readings was ICC > 0.99).

### 2.9. Statistical Analyses

We validated the lateral CDI by examining the Spearman correlations between baseline (month 0) lateral CDI, lateral joint space width (JSW), and static alignment (HKA angle). Scatter plots were generated using the ranking (from smallest to largest) of lateral CDI, JSW, and HKA angle measurements. Tests for trend were used to examine associations of lateral CDI with baseline JSN and KL grade. We calculated standard response mean (SRM) for lateral CDI change between baseline and 24 months. To evaluate the intratester reliability, we calculated intraclass correlation coefficients with a 3,1 model [9].

## 3. Results

### 3.1. Test Dataset (*n* = 80)

We found a good correlation between baseline lateral CDI and lateral cartilage volume (manual segmentation) in this test dataset (lateral femur: spearman correlation = 0.74; lateral tibia: spearman correlation = 0.77; lateral tibiofemoral: *r* = 0.80, all *p* < 0.0001).

### 3.2. Validation Dataset Characteristics (*n* = 100)

The final validation set included 100 knees with a mean age = 64.4 (SD = 9.3) years, 59% females, mean BMI = 28.7 (SD = 4.2) kg/m^2^, mean JSW = 4.4 (SD = 2.3) mm, mean HKA = 3.0° (SD = 4.7°), and a diverse range of baseline lateral JSN grades (0 to 3). The distribution of baseline KL and lateral JSN grades is provided in Table 1. Forty-eight knees had lateral JSN progression over 24 months.

### 3.3. Measurement Time

We recorded the measurement time for the first 10 knees. The average CDI measurement time of 10 knees was 7.4 minutes (SD = 0.73) per pair of knees (baseline and 24-month scans).

### 3.4. Assessment of Reliability

Intratester (ICC (3, 1 model)) reliability for baseline lateral femur, lateral tibia, and total lateral tibiofemoral ranged from 0.86 to 0.98.

### 3.5. Relationship of Lateral CDI to Radiographic Severity

At baseline, knees with greater lateral JSN and KL had lower mean CDI (i.e., greater cartilage damage, Table 1). Baseline lateral femur CDI, baseline lateral tibia CDI, and baseline lateral tibiofemoral CDI are associated with both lateral JSW and static alignment (see Table 2 and Supplementary Figures 1 and 2 in Supplementary Material available online at http://dx.doi.org/10.1155/2015/634275).

### 3.6. Sensitivity to Change

The sensitivity to change is good (SRM = −0.76 for lateral femur; SRM = −0.73 for lateral tibia; SRM = −0.87 for lateral tibiofemoral total).

## 4. Discussion

This study demonstrates that the CDI can be adapted for use in the lateral tibiofemoral compartment by identifying informative locations that are unique to the lateral femur and tibia. This study also shows that the lateral CDI is quick to perform, reliable, and responsive and has good construct validity.

Testing the lateral CDI was important because the lateral denudation regions were in different locations than the medial tibiofemoral compartment [10]. The lateral denudation region is more posterior (both femur and tibia) than medial compartment region. The size of the denudation region is smaller in the lateral compartment compared to the medial compartment.

The lateral CDI had good construct validity relative to other established radiographic measures of knee OA severity and risk factors including lateral tibiofemoral JSN (a semiquantitative scale), lateral JSW (continuous), KL grade (a global semiquantitative score), and knee alignment (continuous). Radiographic JSN and JSW are generally attributed, at least in part, to articular cartilage damage among knees with OA [11]. For example, Bruyere et al. found that lateral tibiofemoral JSW was significantly correlated with baseline lateral tibial cartilage volume (*r* = 0.48, *p* < 0.01) and thickness (*r* = 0.58, *p* < 0.01) [12]. While we only used 18 informative locations, our baseline lateral tibiofemoral CDI had a better correlation with lateral JSW (*r* = 0.81, *p* < 0.0001). We did not look at the correlations with CDI change because Bruyere et al. found that there were no significant correlations between cartilage/thickness loss and lateral JSW [12]. In addition to verifying that the lateral CDI was associated with radiographic OA severity, we also demonstrated that the lateral CDI is related to knee alignment (*r* = −0.30 to −0.33, *p* = 0.004 to 0.01), which is a strong risk factor for knee OA progression [2, 13].

We also found that lateral CDI is sensitive to change over 24 months. One other OAI study found that knees with lateral JSN had more lateral tibiofemoral cartilage loss in 1 year than knees without lateral JSN (SRM = −0.48 versus SRM = −0.09 for total lateral tibiofemoral cartilage thickness change) [14]. Our lateral CDI had a comparable sensitivity (SRM = −0.87 for two-year lateral tibiofemoral change).

The CDI is an efficient method of measuring cartilage damage. The proficient operator can measure the lateral tibiofemoral CDI of a pair of knee MRIs in about 7 minutes. In contrast, the manual MR-based cartilage measurement method may take up to 6 hours per knee [1]. Due to the time and cost of measuring cartilage, most studies only focus on medial tibiofemoral unicompartmental measurements. Using the CDI measurement instead of full manual segmentation represents substantial time and resource savings. Our group plans to complete CDI development to include a comprehensive assessment of knee articular cartilage including medial tibiofemoral, lateral tibiofemoral, and patellofemoral compartments. Such efforts will help develop a quantitative understanding of OA disease progression in a compartment-by-compartment basis.

This study is limited because our validation dataset did not include lateral cartilage segmentation values. However, we found a good correlation between baseline lateral CDI and lateral cartilage volume (manual segmentation) in our test dataset (*r* = 0.74 to 0.80, *p* < 0.0001). Another limitation of CDI is the possibility that the informative locations may not include all cartilage damage. This limitation is similar to other methods that focus on specific articular surface regions [6, 15]. Despite this limitation, we demonstrated that the lateral CDI has good construct validity with radiographic data, which is a common strategy to assess lateral tibiofemoral cartilage data [12, 14, 16].

In summary, the lateral tibiofemoral CDI quantification allows for rapid evaluation and is reliable and responsive, with good construct validity. It may be an efficient method to measure lateral tibiofemoral articular cartilage in large clinical and epidemiologic studies.

## Supplementary Material

Supplemental Figure 1: Plots for lateral CDI and JSW using ranks.
Supplemental Figure 2: Plots for lateral CDI and static alignment (HKA) using ranks.

## Figures and Tables

**Table:** (a) Lateral joint space narrowing (JSN)

Cartilage measure	JSN = 0 (*n* = 25) mean	JSN = 1 (*n* = 25) mean	JSN = 2 (*n* = 25) mean	JSN = 3 (*n* = 25) mean	*p* value for trend
Lateral femur CDI	2969.3	3003.2	2184.4	1542.0	<0.001
Lateral tibia CDI	1154.9	889.9	663.8	392.7	<0.001
Lateral tibiofemoral CDI	4124.3	3893.0	2848.2	1934.6	<0.001

**Table:** (b) Kellgren-Lawrence (KL)

Cartilage measure	KL = 0 (*n* = 10) mean	KL = 1 (*n* = 6) mean	KL = 2 (*n* = 32) mean	KL = 3 (*n* = 26) mean	KL = 4 (*n* = 26) mean	*p* value for trend
Lateral femur CDI	2718.7	2831.5	3061.0	2254.0	1605.3	<0.001
Lateral tibia CDI	1229.3	994.1	946.3	690.4	424.7	<0.001
Lateral tibiofemoral CDI	3948.0	3825.5	4007.3	2944.4	2030.0	<0.001

**Table 2:** Correlation between lateral CDI and baseline HKA and lateral JSW.

	Spearman correlation	
	Lateral JSW	HKA
Femur CDI (baseline)	0.81 (*p* < 0.01)^*∗*^	−0.31 (*p* < 0.01)^*∗*^
Tibia CDI (baseline)	0.81 (*p* < 0.01)^*∗*^	−0.30 (*p* = 0.01)^*∗*^
Tibiofemoral CDI (baseline)	0.85 (*p* < 0.01)^*∗*^	−0.33 (*p* < 0.01)^*∗*^

Notes: ^*∗*^ *p* < 0.05; HKA = hip-knee-ankle; JSW = joint space width.
