# Supplementary material for: Development of a Rapid Cartilage Damage Quantification Method for the Lateral Tibiofemoral Compartment Using Magnetic Resonance Images: Data from the Osteoarthritis Initiative
Source: Biomed Res Int. 2015 Dec 2;2015:634275. doi: 10.1155/2015/634275 (PMC4680059; doi:10.1155/2015/634275)
Supplement: Supplementary file 1 — Supplemental Figure 1: Plots for lateral CDI and JSW using ranks. Supplemental Figure 2: Plots for lateral CDI and static alignment (HKA) using ranks. [file 634275.f1.docx]

Supplemental Figure 1. Plots for Lateral CDI and JSW using ranks. A. Scatter plot of lateral femur CDI ranked according to value and lateral JSW ranked according to value. B. Scatter plot of lateral tibia CDI ranked according to value and lateral JSW ranked according to value. C. Scatter plot of lateral total CDI (femur+tibia) ranked according to value and lateral JSW ranked according to value.


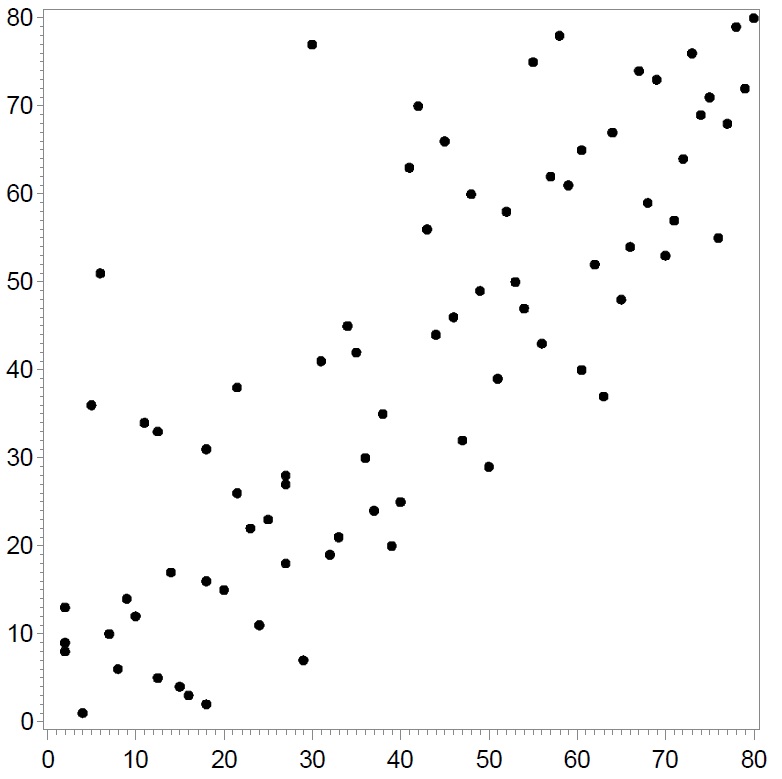

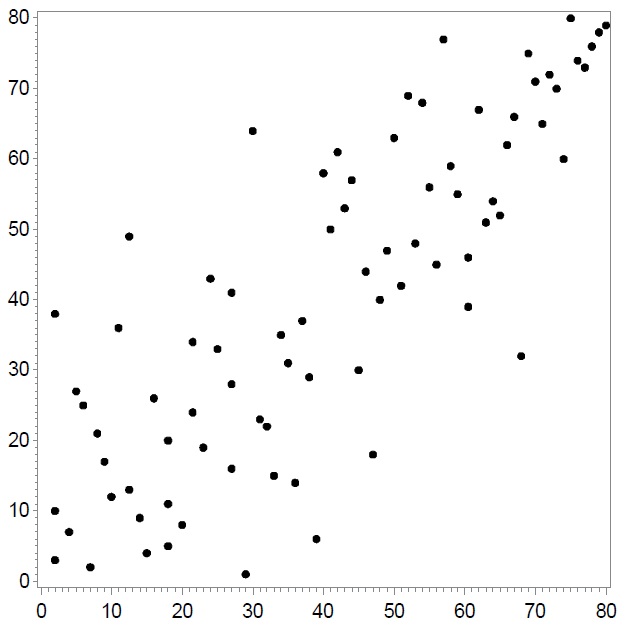

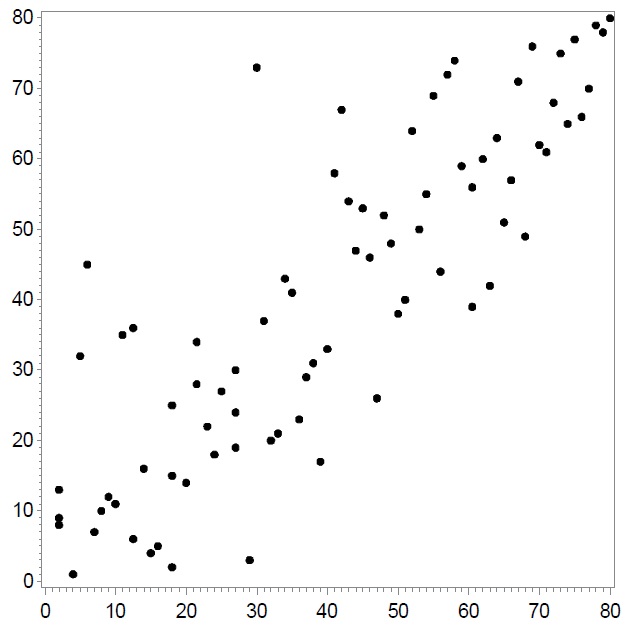


**A**

(r_s_=0.81,p<0.01)

**B**

(r_s_=0.81,p<0.01)

**C**

(r_s_=0.85,p<0.01)

Lateral femur CDI Rank

Lateral JSW Rank

Lateral Tibia CDI Rank

Lateral Total CDI Rank

Lateral JSW Rank

Lateral JSW Rank

**C**

(r_s_= -0.33,p<0.01)

Supplemental Figure 2. Plots for Lateral CDI and static alignment (HKA) using ranks. A. Scatter plot of lateral femur CDI ranked according to value and HKA ranked according to value. B. Scatter plot of lateral tibia CDI ranked according to value and HKA ranked according to value. C. Scatter plot of lateral total CDI (femur+tibia) ranked according to value and HKA ranked according to value.

Lateral femur CDI Rank

HKA Rank

Lateral Tibia CDI Rank

Lateral Total CDI Rank

HKA Rank

HKA Rank


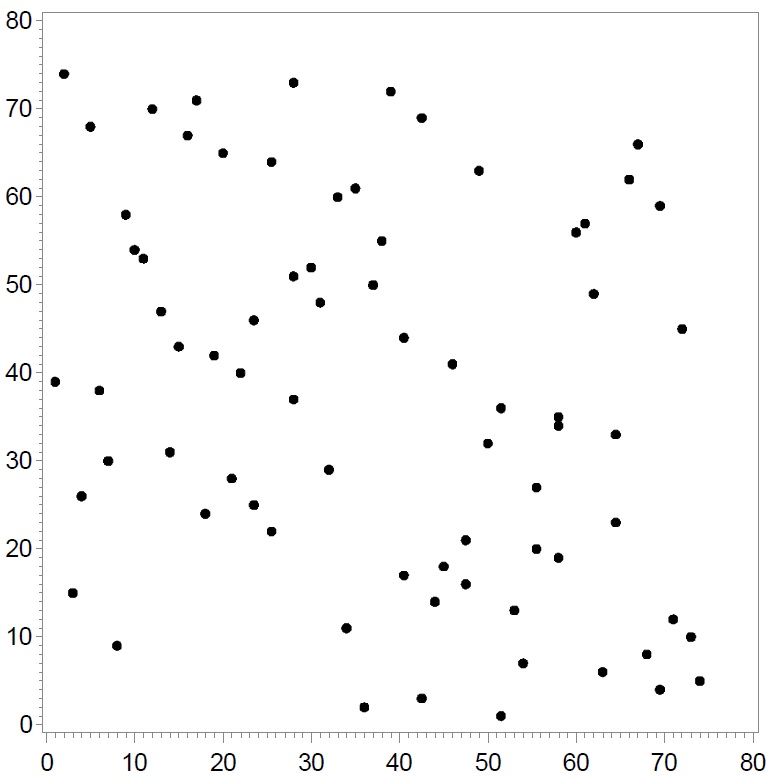

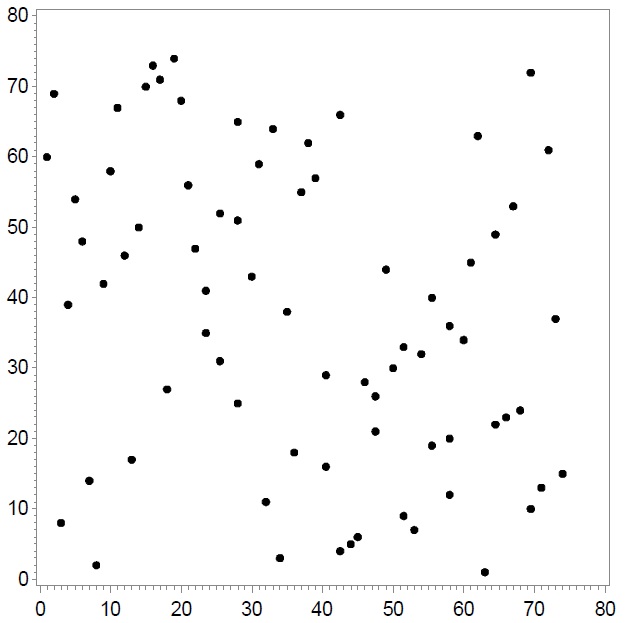


**B**

(r_s_= -0.30,p<0.01)


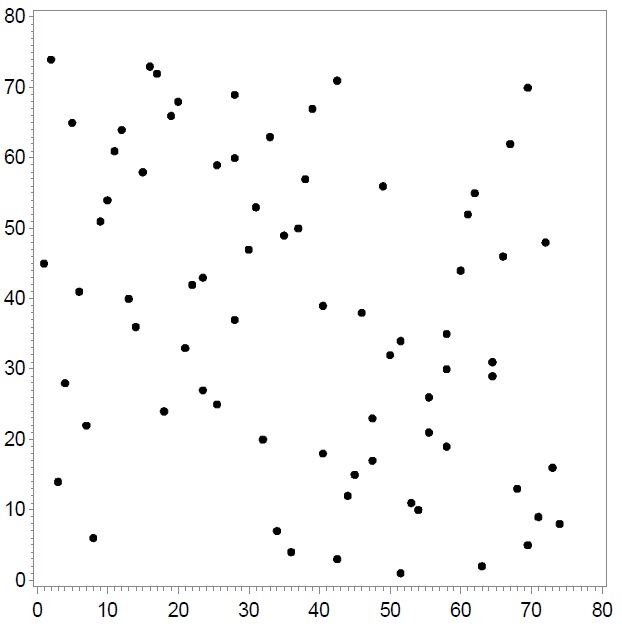


**A**

(r_s_= -0.31,p<0.01)
